# Casein kinase 1α: biological mechanisms and theranostic potential

**DOI:** 10.1186/s12964-018-0236-z

**Published:** 2018-05-24

**Authors:** Shaojie Jiang, Miaofeng Zhang, Jihong Sun, Xiaoming Yang

**Affiliations:** 1 Department of Radiology, Sir Run Run Shaw Hospital, School of Medicine, Zhejiang University, Zhejiang, 310016 Hangzhou China; 2 Department of Orthopaedics, Second Affiliated Hospital, School of Medicine, Zhejiang University, Zhejiang, 310009 Hangzhou China; 3 Image-Guided Bio-Molecular Intervention Research, Department of Radiology, University of Washington School of Medicine, Seattle, WA 98109 USA

**Keywords:** Casein kinase 1α, Wnt/β-catenin signaling, NF-κB signaling, Hedgehog signaling, Autophagy, Neurodegenerative disease, Cell cycle, Host defense response

## Abstract

Casein kinase 1α (CK1α) is a multifunctional protein belonging to the CK1 protein family that is conserved in eukaryotes from yeast to humans. It regulates signaling pathways related to membrane trafficking, cell cycle progression, chromosome segregation, apoptosis, autophagy, cell metabolism, and differentiation in development, circadian rhythm, and the immune response as well as neurodegeneration and cancer. Given its involvement in diverse cellular, physiological, and pathological processes, CK1α is a promising therapeutic target. In this review, we summarize what is known of the biological functions of CK1α, and provide an overview of existing challenges and potential opportunities for advancing theranostics.

## Background

**Fig. 1:**
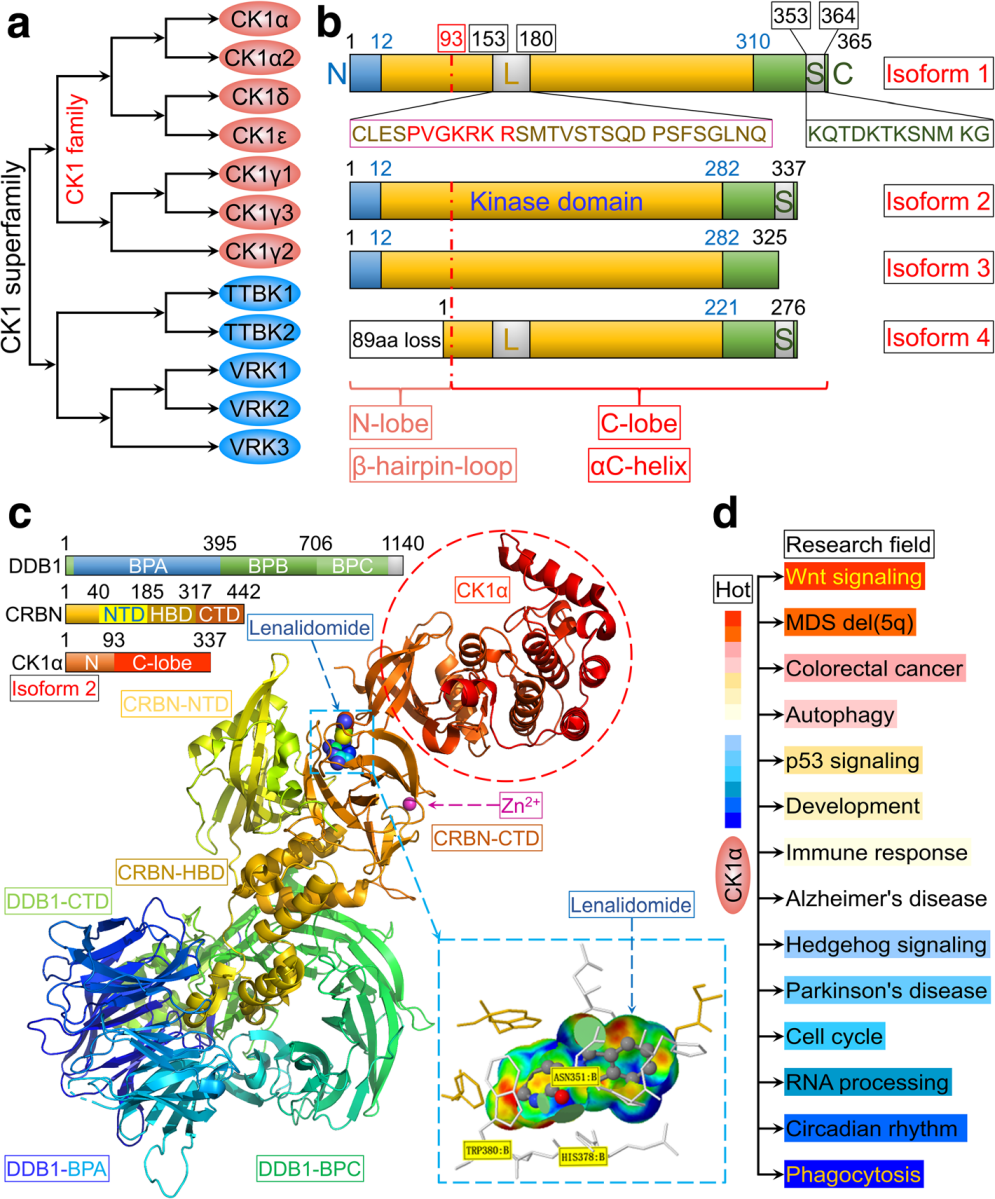
Schematic representation of CK1α. **a** CK1 family and CK1 superfamily. **b** Four isoforms of CK1α and their functional domains. **c** Cartoon representation of DNA damage-binding protein (DDB)1ΔBPB-CRBN-lenalidomide-CK1α. Top left, DDB1, CRBN, and CK1α domain color coding and boundaries. Bottom right, enlarged view of the CRBN-lenalidomide-CK1α interface (data were obtained from protein data bank: www.rcsb.org, PDB-ID: 5FQD; and were first published in reference [13]). **d** Investigations on CK1α in diverse research fields

Casein kinase 1α (CK1α) (encoded by *CSNK1A1* in humans) is a member of the CK1 family of proteins that has broad serine/threonine protein kinase activity [1–4] (Fig. 1a) and is one of the main components of the Wnt/β-catenin signaling pathway. CK1α phosphorylates β-catenin at Ser45 as part of the β-catenin destruction complex for subsequent β-transducin repeat-containing E3 ubiquitin protein ligase (β-TrCP)-mediated ubiquitination and proteasomal degradation [5, 6]. Recent studies have shown that CK1α targets p53 for degradation—which is mediated by murine double minute clone 2 (MDM2) and MDM4 (also known as MDMX) [7–10]—while stabilizing and thereby positively regulating E2F-1, a transcription factor involved in cell cycle progression [7]. Additionally, CK1α was shown to exert dual gating functions by first promoting and then terminating T cell receptor (TCR)-induced nuclear factor κB (NF-κB) activation [11]. Lenalidomide (a thalidomide analog) is a highly effective treatment for myelodysplastic syndrome with deletion of chromosome 5q [MDS del(5q)] that exerts its effects by inducing CK1α ubiquitination and degradation [12, 13]. These findings suggest that *CSNK1A1* is a conditionally essential malignancy gene and a potential target for anti-cancer drugs.

### Overview of CK1α

*CSNK1A1* is located on chromosome 5q32 and is expressed as four alternatively spliced transcript variants, yielding four protein isoforms of varying length that mainly differ by the presence or absence of a 28-amino acid “L” insert in the kinase domain and a 12-amino acid “S” insert near the C terminus. The former is unique to vertebrates [14] and contains the sequence of PVGKRKR, which has the characteristics of a nuclear localization signal (NLS) and may target CK1α to the nucleus [15] (Fig. 1b). Isoform 2, which comprises 337 amino acids, is the predominant isoform [11, 13] with a kinase domain located between Ile12 and Ala282 [11]. The 2.45-Å crystal structure revealed that the first 93 amino acids form a β-hairpin loop and (especially residues 35–41) binds cullin 4/really interesting new gene-box 1/DNA damage-binding protein 1/cereblon (CRBN) (also known as CRL4^CRBN^) E3 ubiquitin ligase for CK1α ubiquitination and degradation [12, 13]. The C-lobe of CK1α is mainly composed of αC helices and contributes to the kinase function (Fig. 1c). CK1α phosphorylates the serine/threonine residue in the canonical motif of pS/T-X_(*n* = 2-4)_-pS/T or noncanonical motif of pS/T-X-pS/T (where pS/T is phospho-serine/threonine and X is any amino acid) [16, 17]. The basic residues (K^229^KQK^232^) of CK1α are implicated in canonical substrate recognition [17], but the noncanonical substrate with pS/T-X-pS/T motif such as β-catenin is not significantly affected by mutations in the K^229^KQK^232^ stretch [17, 18].

CK1α is widely expressed in various organelles including the cell membrane and nucleus [15]. It also localizes to the centrosome, microtubules, the Golgi apparatus, and endoplasmic reticulum in non-neuronal interphase cells [19, 20]; in synaptic vesicles in neurons [20]; spindle microtubules at mitosis [21]; and to nuclear structures (e.g., nuclear speckles) [22]. CK1α is ubiquitously expressed and is constitutively active [23, 24], implying that it has many biological functions besides its role in β-catenin degradation that span diverse research areas (Fig. 1d).

### Physiological and pathological expression of CK1α in humans

**Fig. 2:**
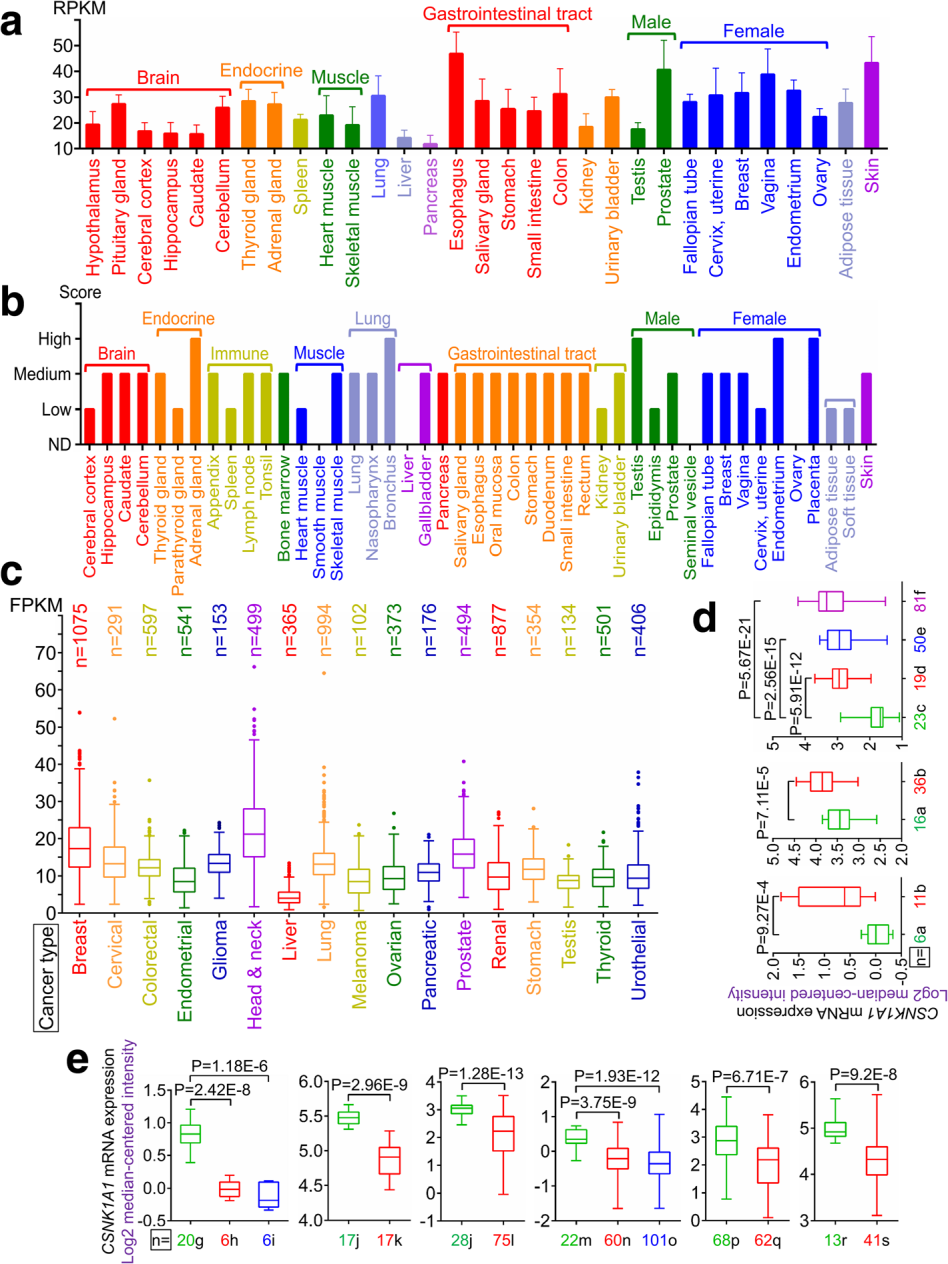
CK1α expression in normal human tissues and the most common human cancer tissues. **a** RNA sequencing data for CK1α expressed in normal human tissues are reported as median reads per kilobase per million mapped reads (RPKM). The data were generated by the Genotype-Tissue Expression project (www.gtexportal.org) and were first published in references [238, 239] and deposited in the HPA (www.proteinatlas.org). **b** Protein expression data from HPA (www.proteinatlas.org), first published in reference [240]. **c** RNA sequencing data of CK1α levels in 17 cancer types are reported as median number of fragments per kilobase of exon per million reads (FPKM), generated by The Cancer Genome Atlas (TCGA) (https://cancergenome.nih.gov/); data were first published in reference [241], and were deposited in the HPA (www.proteinatlas.org). **d**, **e** Microarray data of CK1α expression in normal and cancer tissues in humans were obtained from Oncomine (www.oncomine.org) (reference [242]). Differences in expression levels were evaluated with the Student’s t test using Oncomine software. **d** Upregulation of CK1α mRNA levels in human cancer tissues relative to matched normal tissues. a, Pancreas, b, pancreatic carcinoma (left, reference [243] and right, reference [244]); c, brain; d, anaplastic astrocytoma; e, oligodendroglioma; f, glioblastoma (reference [245]). **e** Downregulation of CK1α mRNA levels in human cancer tissues relative to matched normal tissues. g, CD4-positive (*n* = 5) + CD8-positive (*n* = 5) + normal T lymphocytes (*n* = 10); h, angioimmunoblastic T-cell lymphoma; i, anaplastic large cell lymphoma (reference [246]); j, esophagus; k, esophageal squamous cell carcinoma; l, esophageal adenocarcinoma (left, reference [247]; right, reference [248]); m, colon (*n* = 19) + rectum (*n* = 3); n, rectal adenocarcinoma; o, colon adenocarcinoma (data obtained from TCGA and deposited in Oncomine); p, bladder mucosa; q, infiltrating bladder urothelial carcinoma (reference [249]); r, buccal mucosa; s, head and neck squamous cell carcinoma (reference [250])

**Fig. 3:**
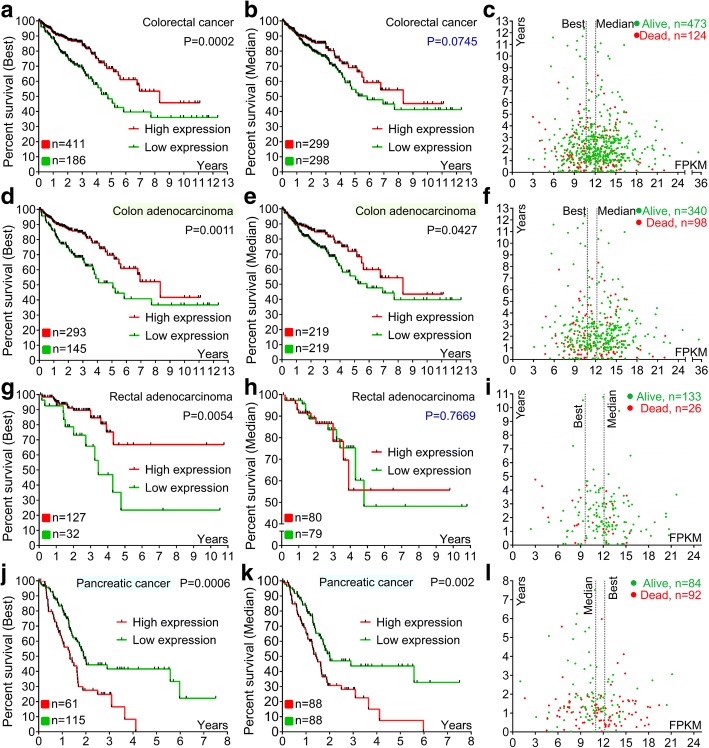
Prognostic value of CK1α mRNA level in human colorectal and pancreatic cancers. Data were obtained from The Cancer Genome Atlas and deposited in the HPA (www.proteinatlas.org). *P* values were estimated with the Kaplan-Meier method. **a**, **b**, **d**, **e**, **g**, **h**, **j**, **k** Kaplan-Meier survival analysis of colorectal cancer, colon adenocarcinoma, rectal adenocarcinoma, and pancreatic cancer by best (left) and median (right) separation according to CK1α mRNA expression level. **c**, **f**, **i**, **l** Interactive survival plot (individual patient data)

**Fig. 4:**
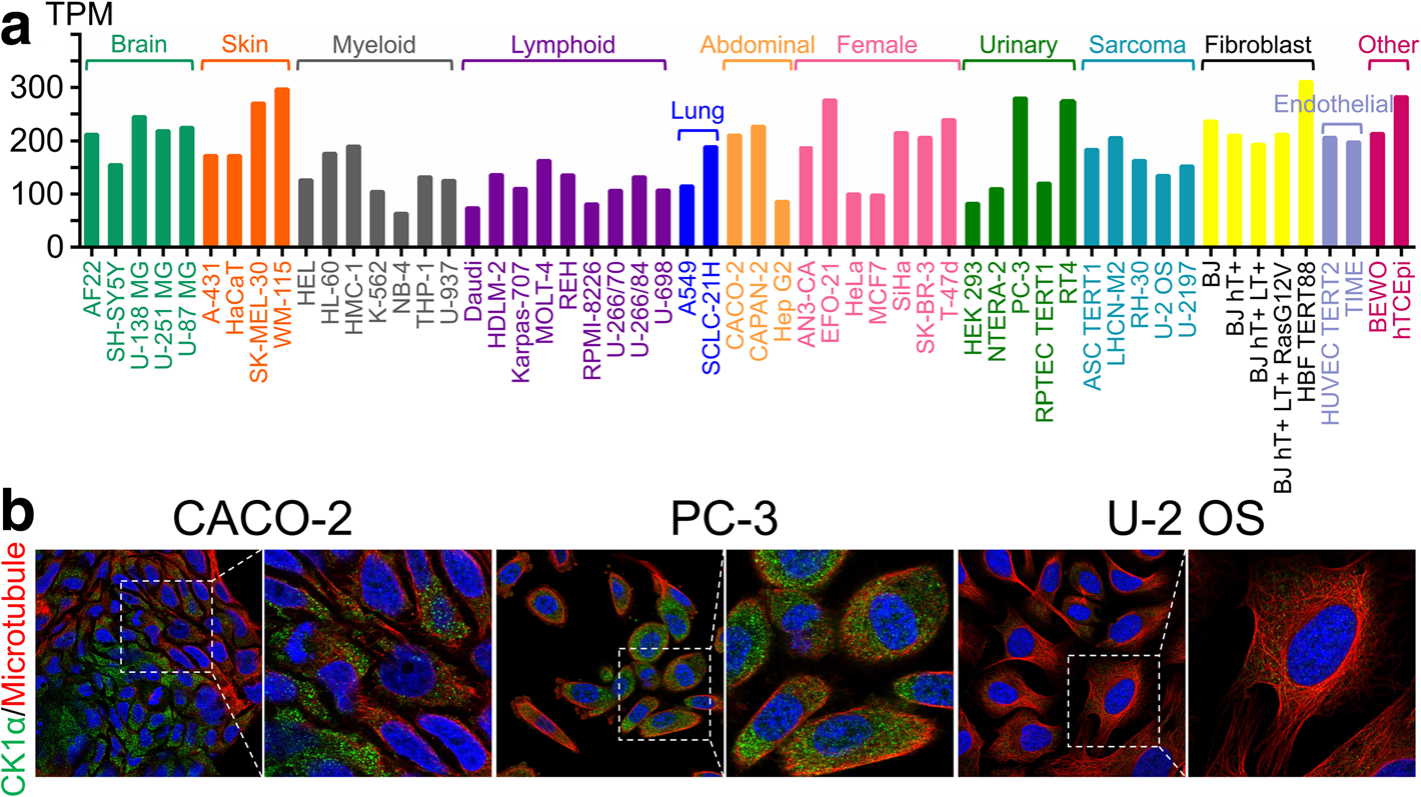
CK1α mRNA and protein expression in common cell lines. **a** RNA sequencing data for CK1α from the HPA (www.proteinatlas.org) are reported as number of transcripts per kilobase million. **b** Subcellular localization of CK1α in Caco-2, PC-3, and U-2 OS cell lines. Data were obtained from the HPA (www.proteinatlas.org) and were first published in reference [251]

CK1α mRNA is expressed in all tissues in humans under physiological conditions; the levels are high in esophagus and skin, but low in pancreas and liver (Fig. 2a). The protein is highly expressed in adrenal gland, bronchus, testis, placenta, and endometrium but is not detected in smooth muscle, liver, seminal vesicle, or ovary (Fig. 2b). CK1α mRNA is expressed in most cancer tissues (Fig. 2c), and highly expressed in pancreatic cancer but is detected at low levels in colorectal cancer as compared to matched normal tissues with GeneChip arrays (Fig. 2d, e). Interestingly, low CK1α expression was associated with poorer overall survival (OS) in colorectal cancer patients (Fig. 3a–c), especially in colon adenocarcinoma (Fig. 3d–i). On the other hand, high CK1α levels in pancreatic cancer were linked to poorer OS (Fig. 3j–l), providing evidence that CK1α is a conditionally essential malignancy protein. CK1α mRNA was also found to be expressed in various cancer cell lines (Fig. 4a) and was localized to the cytosol (Fig. 4b), suggesting that it mainly functions in the cytoplasm.

### CK1α in Wnt/β-catenin and hedgehog signaling

Wnt/β-catenin (also known as canonical Wnt) signaling regulates various physiological processes including embryonic development, adult stem cell maintenance, and genomic stability [25]. Mutations in Wnt pathway components such as adenomatous polyposis coli (APC) result in pathological disturbances, especially in colorectal cancer [26]. β-catenin is a key component of this pathway that binds to the cytoplasmic tail of E-cadherin at the cell membrane to promote cell-cell adhesion [27], and also localizes to the cytoplasm where it forms the destruction complex along with CK1α, glycogen synthase kinase 3β (GSK-3β), APC, Axin, and Wilms tumor gene on X chromosome (WTX, also known as APC membrane recruitment protein 1) to promote the ubiquitination and proteasomal degradation of β-catenin in the absence of extracellular Wnt ligands [28]. β-Catenin is translocated to the nucleus upon activation of Wnt signaling via Rac1 [29], where it forms a complex with T cell factor and co-activators such as cyclic (c)AMP response element-binding protein (CREB)-binding protein and BRM/SWI2-related gene 1 (Brg-1) to activate Wnt target genes [30].

β-Catenin is phosphorylated by CK1α at Ser45, which leads to GSK-3β-dependent phosphorylation at Ser33/37 and Thr41 and subsequent degradation [5]. APC is also phosphorylated at Ser1504/1505/1507 and S1510 (in the R3 region) by CK1α and other CK1 proteins [31], which is essential for β-catenin binding. Thus, CK1α acts as a negative regulator of Wnt signaling [32].

**Fig. 5:**
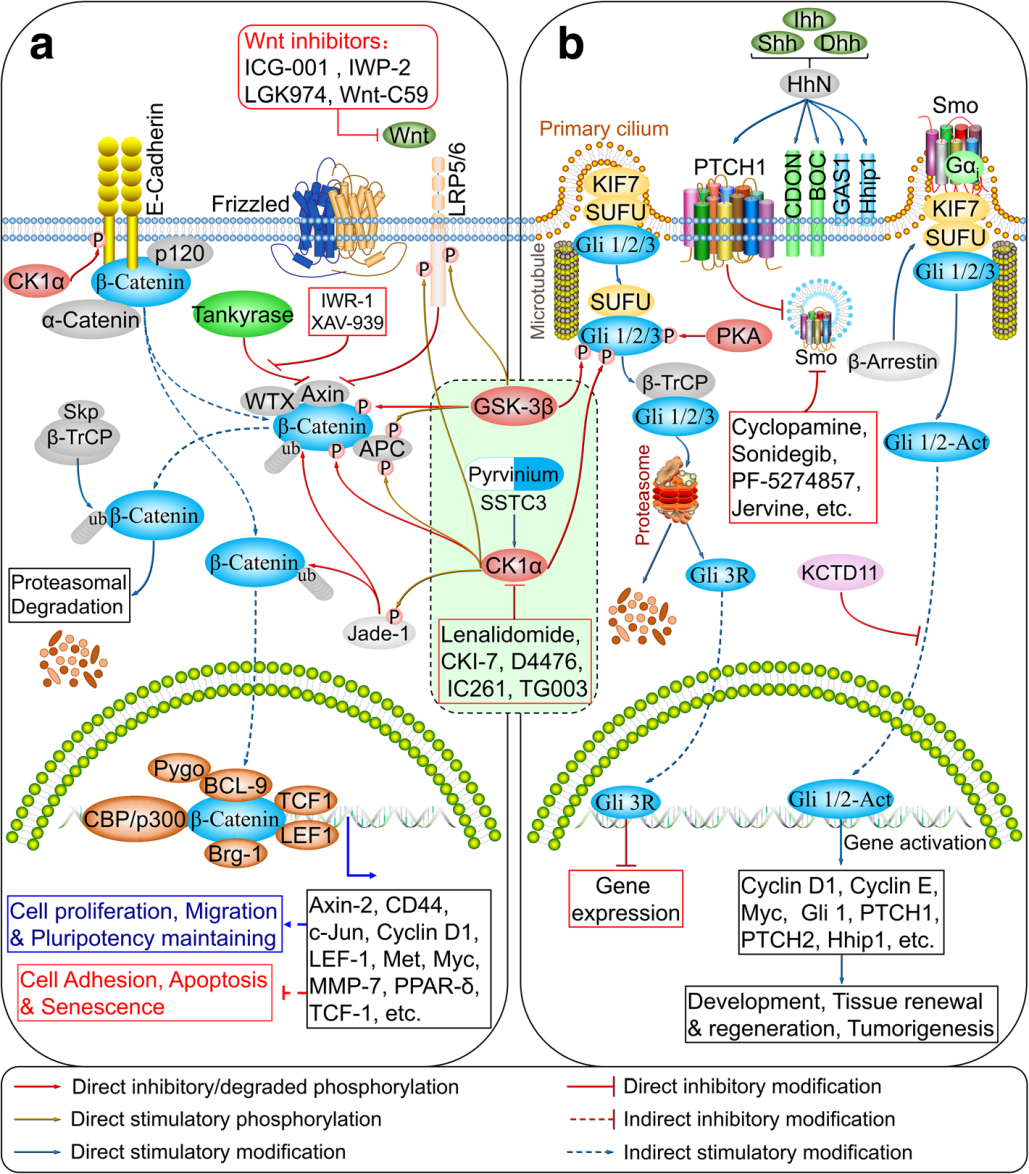
CK1α mediates crosstalk between Wnt/β-catenin and Hedgehog signaling networks. **a**, **b** CK1α in Wnt/β-catenin (**a**) and Hedgehog (**b**) signaling pathways. (also reviewed in references [41, 252])

The cytoplasmic domain of E-cadherin is phosphorylated by CK1α at Ser846, which attenuates its interaction with while promoting the release of β-catenin from the cell membrane [33]. Low-density lipoprotein receptor-related protein 6 (LRP6) is a single-pass transmembrane receptor that cooperates with Frizzled proteins for Wnt ligand binding and can be phosphorylated by CK1α and CK1δ at Thr1493, which activates and promotes recruitment of Axin to the membrane in response to the Wnt signal, leading to Wnt pathway activation [34]. The plant homeodomain zinc finger protein Jade-1 functions as an E3 ubiquitin ligase that ubiquitinates both phosphorylated and non-phosphorylated forms of β-catenin [35] and is a substrate of CK1α; it is phosphorylated at Ser18 and Ser20, which reduces its ability to inhibit Wnt/β-catenin signaling [36, 37]. Thus, CK1α can act as a positive regulator of Wnt/β-catenin signaling (Fig. 5a and Table 1).

**Table 1:** Substrates of human CK1α in major cell signaling pathways

Gene	Protein	Phosphorylation site	Pathway	Reference
APC	APC	S1504, S1505, S1507, S1510	Wnt/β-Catenin	[31]
CTNNB1	β-Catenin	S45		[5, 6]
CDH1	E-cadherin	S846		[33]
LRP6	LRP6	T1493		[34]
JADE1	JADE1	S18, S20		[36, 37]
FOXO3A	FOXO3A	S318, S321	Autophagy	[72]
DEPTOR	DEPTOR	S286, S287, S291		[74, 75]
SQSTM1	SQSTM1/p62	S349		[79]
BCL10	BCL10	N/A	NF-κB	[11, 260]
CARD11	CARMA1	S608		[11, 260]
MALT1	MALT1	N/A		[11, 260]
FADD	FADD	S194		[80, 86]
RELA	NF-κB/p65	S316		[261]
RIPK1	RIP1	a.a. 293–558		[83]
TNFRSF1A	TNFR1/p55	N/A		[83]
TNFRSF1B	TNFR2/p75	N/A		[84]
YBX1	YB-1	S176		[89]
CDC25A	CDC25A	S79, S82	Cell cycle	[92, 93]
MDM2	MDM2	N/A		[7, 95]
MDM4	MDMX	S289		[8, 9, 96]
MYC	c-Myc	S252		[94]
TP53	P53	S20		[97]
YWHAQ	14-3-3τ	S233		[101]
YWHAZ	14-3-3ζ	T233		[101]
BACE1	β-Secretase 1	S498	Alzheimer’s disease	[113]
KCNIP3	Calsenilin	S63		[115]
CREB1	CREB	S108, S111, S114	Parkinson’s disease	[128]
LRRK2	PARK8	S910, S935, S955, S973		[131]
PARK2	Parkin	S101, S378		[132]
SNCA	α-Synuclein	S87, S129		[126]
CDK5	CDK5	S159		[134]

The development of the Cre-LoxP system has enabled detailed investigations of the opposing functions of CK1α in Wnt signaling. For example, gut-specific knockout of CK1α using the Villin 1 promoter resulted in Wnt hyperactivation due to decreased phosphorylation of β-catenin at Ser45, Ser33/37, and Thr41 and an increment in total β-catenin levels. Accordingly, target genes of Wnt signaling such as cyclin D1, c-myc, and CD44 were induced at both the mRNA and protein levels in CK1α knockout mice [10]. Reporter-based screens of haploid human cells revealed that CK1α and APC were the rate-limiting negative regulators of Wnt signaling [38].

Hedgehog signaling is aberrantly activated in basal cell carcinomas, the most common cancer in humans [39] and in medulloblastoma, the most common pediatric brain malignancy [40]. Gli transcription factors are key mediators of Hedgehog signaling and are phosphorylated by CK1α, GSK-3β, and protein kinase A (PKA), which promote the proteolysis of the active form of Gli1/2 and induction of a repressive form of Gli3 receptor [41]. In *Drosophila*, CK1α suppresses Hedgehog signaling in the absence of a ligand [42, 43] and is also required for Smoothened (Smo) phosphorylation upon pathway activation [44–48]. However, Smo in mammals lacks CK1α phosphorylation sites [47].

Hedgehog signaling shares many components with the Wnt/β-catenin pathway, including CK1α, GSK-3β, and β-TrCP [49, 50]. Pyrvinium, a CK1α agonist that is known to block Wnt signaling [51], suppresses Hedgehog signaling by attenuating Gli activity [52]. Thus, CK1α functions as a negative regulator of Hedgehog signaling in mammals (Fig. 5b).

### CK1α in the regulation of autophagy

Autophagy plays an important role in the maintenance of organismal homeostasis through regulation of cellular protein and organelle turnover, with their subsequent degradation by lysosomes providing macromolecular precursors and energy to cells [53]. Aberrant autophagy leads to various diseases such as cancer and neurodegeneration [54]. Autophagy is an evolutionarily conserved catabolic process that has five distinct stages: initiation, vesicle nucleation, vesicle elongation, vesicle fusion, and cargo degradation [54]. It is induced by nutrient deficiency, oxidative stress, and infection, among other factors. Vesicle nucleation is induced by an activated Unc-51-like autophagy activating kinase 1 (ULK1) complex, which consists of ULK1/2 (ortholog of yeast autophagy-related 1 [Atg1]), focal adhesion kinase family interacting protein of 200 kDa (ortholog of yeast Atg17) [55], Atg13, and Atg101 [56, 57], which is released from mammalian target of rapamycin (mTOR) inhibition [58]. Beclin-1 is then phosphorylated by ULK1 and serves as a scaffold for the class III phosphatidylinositol-3 kinase (PI3K) complex, promoting the localization of autophagy proteins to the phagophore [59]. During this process, autophagy and Beclin-1 regulator 1 binds to Beclin-1 (ortholog of yeast Atg6) to stabilize the PI3K complex, while Barkor (ortholog of yeast Atg14), ultraviolet radiation resistance-associated gene protein, and p150 (ortholog of yeast vacuolar protein sorting-associated protein 15 [Vps15]) bind to Beclin-1 to promote its interaction with Vps34 and phagophore formation [59–64]. Vesicle elongation is mediated by Atg12–Atg5 [65] and microtubule-associated protein 1A/1B-light chain 3-II (LC3-II) [66] along with LC3-like molecules such as gamma-aminobutyric acid type A receptor-associated proteins (GABARAPs) [67], leading to the formation of an autophagosome. Atg12–Atg5 conjugation is mediated by the E1-like enzyme Atg7 and E2-like enzyme Atg10 [65], while LC3B (ortholog of yeast Atg8) is cleaved at the C terminus by Atg4B protease to generate cytosolic LC3-I, which is conjugated to phosphatidylethanolamine by Atg7–Atg3, yielding lipidated LC3-II [66]. Finally, syntaxin 17 facilitates autophagosome fusion with the lysosome for autophagolysosome formation [68], with the cargo then degraded under low-pH conditions (also reviewed in references [53, 54]).

Among the above-mentioned autophagy-related genes, LC3B, GABARAPs (including GABARAP, GABARAPL1, and GABARAPL2), Atg4B, Atg12, and ULK2 were shown to be directly regulated by the transcription factor Forkhead box protein O3A (FOXO3A) [69–71], which is a CK1α substrate that is phosphorylated at Ser318 and Ser321. Treatment with the CK1 inhibitor D4476 or short interfering RNA siRNA-mediated knockdown of CK1α results in nuclear accumulation of FOXO3A and increased expression of autophagy-related genes. CK1α protein abundance is regulated by PI3K/mTOR signaling induced by activated or oncogenic/mutant RAS [72]. DEP domain-containing mTOR-interacting protein (DEPTOR), an mTOR inhibitor [73], is also phosphorylated by CK1α at Ser286/287/291 after priming phosphorylation by mTOR for subsequent degradation mediated by β-TrCP [74–76]. CK1α inhibition by D4476 or siRNA treatment results in upregulation of DEPTOR followed by suppression of mTOR signaling and induction of autophagy [75, 76]. CK1α is a key modulator of autophagic flux, and *CSNK1A1* knockout mediated by transcription activator-like effector nucleases accelerated the turnover of long-lived proteins [77]. A similar observation was made in a previous study demonstrating that *CSNK1A1* knockdown strongly induced autophagic flux [78]. Thus, CK1α negatively regulates autophagy.

**Fig. 6:**
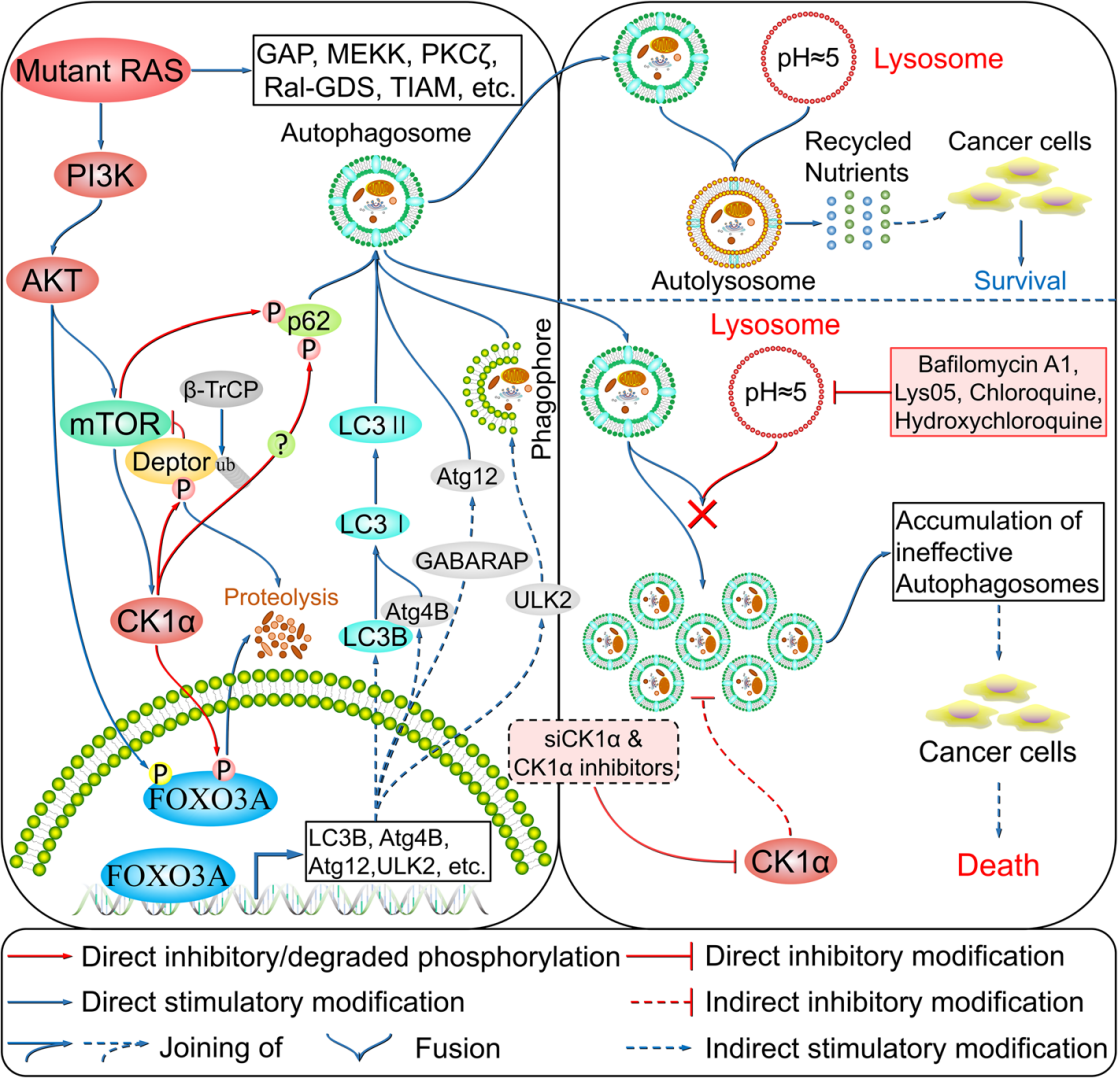
Regulation of autophagy by CK1α. (also reviewed in references [54, 72])

Sequestosome 1 (SQSTM1) (also known as p62)—an autophagy adaptor/receptor and LC3-binding protein that targets specific substrates to autophagosomes [53]—is also phosphorylated by CK1 isoforms at Ser349 upon accumulation of dysfunctional proteins. Phosphorylated SQSTM1 accelerates the formation of inclusion bodies and autophagic protein clearance [79]. However, the induction of autophagy by CK1-mediated phosphorylation of SQSTM1 requires confirmation by co-immunoprecipitation and loss-of-function studies. The combination of CK1α suppression and treatment with lysosome inhibitors such as chloroquine leads to accumulation of ineffective autophagosomes that deprive cancer cells of nutrients required for growth, resulting in their death [72]. CK1α therefore is a promising target for drugs that can be used in combination with lysosome inhibitors, especially in RAS-driven and mTOR-activated cancers [72, 80] (Fig. 6 and Table 1). Notably, there is a discrepancy in the action modes of CK1α in non-small-cell lung cancer (NSCLC) versus RAS-driven colon cancer. CK1α overexpression potently induces autophagic flux in NSCLC via the PTEN/AKT/FOXO3A/Atg7 axis. It stabilizes phosphatase and tensin homolog deleted on chromosome ten (PTEN) by abrogating PTEN phosphorylation and antagonizing neural precursor cell expressed, developmentally down-regulated 4-1 (NEDD4-1) induced PTEN polyubiquitination, which suppresses NSCLC cell growth [81]. CK1α exhibits dual functions in autophagy regulation based on these evidences.

### CK1α in NF-κB signaling

NF-κB signaling is a complex signaling pathway involved in innate and adaptive immunity, inflammation, lymphocyte development, and lymphoid organogenesis, and includes the components NF-κB (RelA/p65), NF-κB1 (p105/p50), NF-κB2 (p100/p52), RelB, and c-Rel [82]. NF-κB signaling is activated by various extracellular ligands and their receptors—e.g., tumor necrosis factor receptor (TNFR), interleukin (IL)-1 receptor, Toll-like receptors, B cell receptor (BCR), and TCR. This activates the inhibitor of κB kinase (IKK) complex (IKKα, IKKβ, IKKγ/NF-kappa-B essential modulator), which phosphorylates inhibitor of κBs (IκBs) and targets them for ubiquitination and proteasomal degradation. The free NF-κB/Rel complex is then modified by a series of kinases and translocated to the nucleus, where its activation alone or in combination with other transcription factors induces the expression of target genes.

**Fig. 7:**
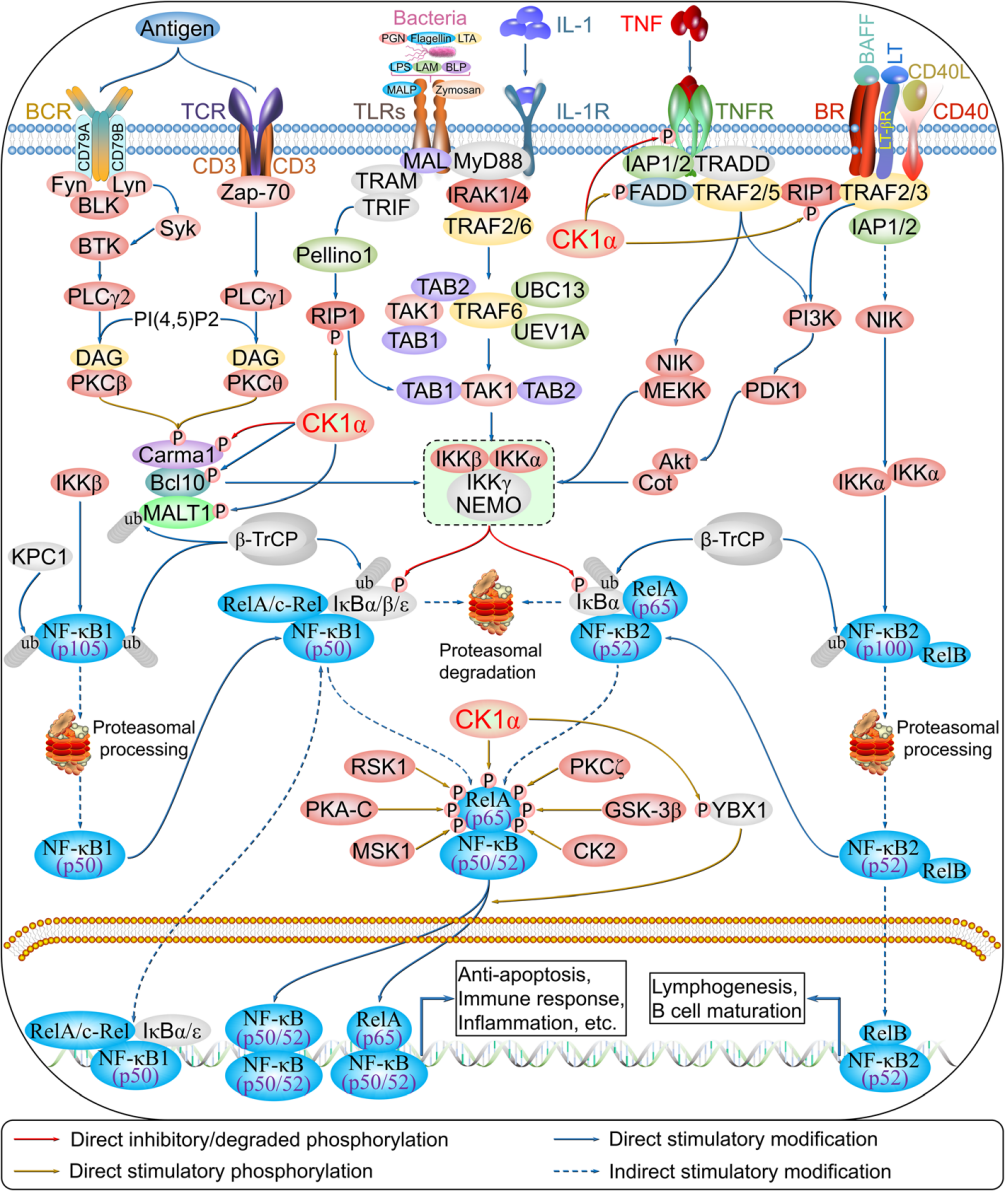
Regulation of NF-κB signaling by CK1α. (also reviewed in references [253–257])

TNF-α is a pro-inflammatory cytokine that activates two distinct cell surface receptors—namely, TNFR1 (p55) and TNFR2 (p75). CK1α binds to and phosphorylates TNFR1 and TNFR2, which negatively regulate TNF-α-mediated NF-κB activation [83, 84]. Receptor-interacting serine/threonine kinase 1 (RIP1) is a critical factor in programmed necrosis, but also mediates TNF-α activation of NF-κB [85]. However, RIP1 is phosphorylated by CK1α at amino acids 293–558, which potentiates TNF-α-mediated NF-κB activation [83]. These two opposing activities suggest that NF-κB signaling regulates CK1α, which also exhibits dual functions in immunoregulation. Fas-associated death domain (FADD) is an adaptor protein that transmits apoptotic signals through death receptors; it directly binds to RIP1, and mediates both necrosis and NF-κB activation. CK1α phosphorylates FADD at Ser194 [80, 86], which is essential for NF-κB activation [87]. The caspase recruitment domain family member 11 (CARD11)/B-cell chronic lymphocytic leukemia/lymphoma 10 (BCL10)/mucosa-associated lymphoid tissue lymphoma translocation gene 1 (MALT1) (CBM) signalosome complex functions as an adaptor to activate IKKs in antigen-receptor-induced NF-κB activation. Notably, CK1α has been shown to directly bind to the CBM complex leading to NF-κB activation in response to TCR stimulation in normal lymphocytes, which largely depends on the association of phosphorylated BCL10 and ubiquitinated MALT1 with CK1α. Inhibitory phosphorylation of caspase recruitment domain-containing membrane-associated guanylate kinase protein 1 at Ser608 by CK1α impairs its ability to activate NF-κB. Activated B cell-like subtype of diffuse large B-cell lymphoma (ABC DLBCL) cells require CK1α for constitutive NF-κB activity [11, 88]; additionally, the oncoprotein Y box-binding protein 1 is phosphorylated by CK1α at Ser176, resulting in NF-κB activation [89]. These findings provide evidence that CK1α has dual functions in NF-κB signaling (Fig. 7 and Table 1).

### CK1α in cell cycle regulation

The mammalian cell cycle is a highly organized and regulated process initiated by mitogenic, growth, or survival signals [90] that activate downstream signaling pathways including mitogen-activated protein kinase signaling and induce the transcription of early-response genes including Myc, activator protein 1, β-catenin, c-Fos, and c-Jun. These in turn activate the expression of delayed-response genes including E2F1, cyclin D-cyclin-dependent kinase 4/6 (CDK4/6, also known as G1-CDK) complex, and cyclin E-CDK2 (also known as G1/S-CDK) complex. Cell division cycle 25 homolog A (CDC25A) potentiates the activity of G1- and G1/S-CDK to promote G1-S transition; G1/S-CDK then inactivates cyclin-dependent-kinase inhibitors (CKIs) by phosphorylation and removes the inhibition of the cyclin A-CDK2 complex (also known as S-CDK). The pre-replication complex is phosphorylated by S-CDK and dissociates to ensure duplication of genetic material and cell division. During G2 phase, the multi-vulval class B (MUVB) complex associates with forkhead box M1 (FOXM1), which binds to promoters containing a cell cycle genes homology region (CHR). This induces the transcription of genes required for G2-M cell cycle transition such as cyclin B-CDK1 (also known as M-CDK), which is activated by CDC25 family members that dephosphorylate Thr14 and Tyr15 via membrane-associated tyrosine/threonine 1 (MYT1, also known as PKMYT1) and WEE1, respectively. Meanwhile, CDK1 is phosphorylated at Thr161 by the cyclin H-CDK7 complex, leading to M phase entry.

CK1α exhibits cell cycle-dependent subcellular localization, including association with cytosolic vesicles and the nucleus during interphase and with the spindle during mitosis [20, 21, 91]. As stated above, β-catenin is a substrate of CK1α, and early-response genes including Myc and c-Jun are targets of Wnt/β-catenin signaling. CK1α also phosphorylates CDC25A at Ser79 and Ser82, which stimulates the binding of β-TrCP for subsequent ubiquitin-mediated proteolysis [92, 93]. Additionally, c-myc is phosphorylated by CK1α at Ser252 through glioma pathogenesis-related protein 1 (GLIPR1) regulation, which is critical for its degradation [94]. Thus, CK1α functions as a negative regulator in the early stages of the G1-S transition.

MDM2 and MDM4 together inhibit DNA binding and transcriptional activation of p53. Inhibition or knockdown of CK1α was shown to increase p53, MDM2, and p21 levels and lead to dephosphorylation of RB, an inhibitor of the G1-S transition [7]. It was later confirmed that treatment with D4476 triggered an increase in nuclear p53 protein level, although the upregulation of MDM2 was mainly cytoplasmic rather than nuclear [95]. This implies that CK1α interacts with MDM2 to stimulate its binding to p53, leading to ubiquitination and degradation of the latter. Moreover, MDMX is phosphorylated by CK1α at Ser289, which is necessary for the MDMX–p53 interaction and inhibition of the DNA-binding and transcriptional activity of p53 [8, 9, 96]. Thus, CK1α is a positive regulator of the G2-M transition.

**Fig. 8:**
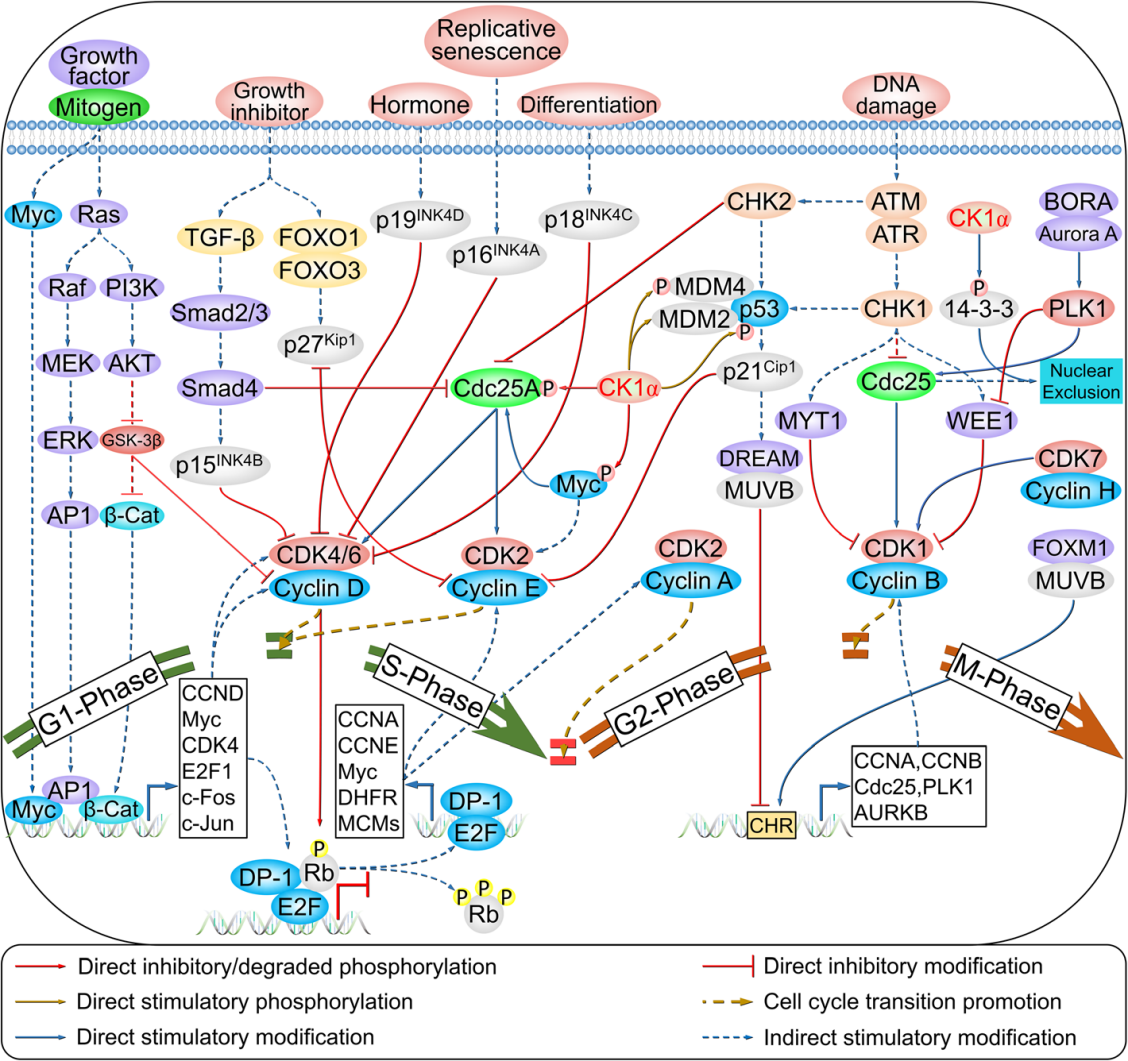
Cell cycle regulation by CK1α. (also reviewed in reference [90])

p53 is directly phosphorylated by CK1α at Ser20 upon infection with human herpesvirus 6B viral [97]. Additionally, the Ser20 residue of p53 is phosphorylated by checkpoint kinase 1/2 in response to DNA damage, which enhances its tetramerization, stability, and activity [98, 99]. To date, there is no in vivo or in vitro evidence for direct phosphorylation of p53 at Ser15 by CK1α; however, this is thought to occur through regulation of F-box and WD repeat domain-containing 7 (FBXW7), which influences the cell cycle and drug resistance [100]. CK1α also phosphorylates 14-3-3τ and 14-3-3ζ at Ser23 and Thr233, respectively [101], thereby modulating their interaction with and nuclear exclusion of M-CDK (Fig. 8 and Table 1).

Jade-1 phosphorylation by CK1α and polo-like kinase 1 (PLK1) is an important biological event for cell cycle progression that involves phosphorylated FADD, which is most abundant during the G2/M phase. CK1α colocalizes with its substrate FADD, which is phosphorylated at Ser194 in metaphase and early anaphase. Suppression of kinase activity by CKI-7 or siRNA-mediated CK1α knockdown abrogates G2/M arrest induced by taxol [80, 86].

Less is known about the function of CK1α in meiosis. CK1α localizes to the spindle poles, which may not be required for meiotic progression in mammalian oocytes since RNA interference (RNAi) or overexpression of CK1α results in invalid spindle organization and chromosome segregation [102]. CK1α is activated in fertilized mouse oocytes but not in metaphase II-arrested mouse oocytes. Microinjection of a blocking antibody against CK1α during metaphase II arrest and G2 phase had no effect on the completion of the second meiosis or first division; however, injection during the early pronuclear stage prior to S phase blocked kinase entry into pronuclei and interfered with timely cell cycle progression to the first cleavage [91]. However, another study showed that CK1α was upregulated in metaphase and colocalized with condensed chromosomes during oocyte maturation and embryonic development; blocking CK1α resulted in the failure of polar body 1 (PB1) extrusion, chromosome misalignment, and metaphase II plate incrassation, while activating CK1α by pyrvinium pamoate treatment inhibited oocyte meiotic maturation and caused severe abnormalities in congression and chromosome misalignment [103].

Suppression of CK1α in the gut triggers Wnt hyperactivation but does not lead to tumorigenesis, since the DNA damage response and cellular senescence are activated via induction of p53 and its downstream effector p21 [10]. Notably, *CSNK1A1* deficiency caused hematopoietic stem cells (HSCs) to exit quiescence and re-enter the cell cycle; meanwhile, *CSNK1A1* haploinsufficiency induced HSCs expansion and increased the S/G2/M-phase fractions, whereas homozygous deletion induced significant induction of early and late apoptosis and led to HSCs failure [104]. CK1α loss was associated with cell cycle arrest in human colorectal polyps [105], and inhibition of CK1α kinase activity in multiple myeloma cells by D4476 or siRNA treatment triggered G0/G1 arrest, prolonged G2/M phase, and increased apoptosis [106]. These findings indicate that CK1α has dual functions in cell cycle progression and cell division.

### CK1α in neurodegenerative diseases

Alzheimer’s disease (AD) is a progressive neurologic disease and leading cause of dementia that is characterized by the irreversible loss of neurons—particularly in the cortex and hippocampus [107]—leading to memory disorder, personality changes, and cognitive dysfunction [108]. Additional histopathological hallmarks include the presence of extracellular senile plaques containing the amyloid-β (Aβ) peptides and neurofibrillary tangles (NFTs) [107].

Aβ peptides are generated by the sequential cleavage of Aβ precursor protein (APP). In a normal state, the Aβ domain of APP is cleaved by α-secretases (mainly A disintegrase and metalloprotease 10 [ADAM10]), releasing soluble N-terminal (s)APPα and C-terminal fragment α (CTFα). The latter is cleaved by the γ-secretase complex composed of catalytic presenilin 1/2 (PS1/2), nicastrin (NCT), PS enhancer 2 (PEN2), and anterior pharynx defective 1/2 (APH1/2), yielding a soluble extracellular p3 peptide and the APP intracellular domain (AICD). When the amyloidogenic pathway is activated in AD, APP is cleaved by β-secretase 1/2, which releases the ectodomains sAPPβ and CTFβ; subsequent cleavage of CTFα by γ-secretase yields Aβ and AICD [109, 110]. CK1 isoforms are upregulated in the brain of AD patients [111, 112] and directly phosphorylate β-secretase at Ser498, thereby regulating trafficking of β-secretase in the secretory and endocytic pathways [113]. Calsenilin (CSEN) binds PS1/2, the catalytic core of γ-secretase complex, and regulates its APP cleavage activity [114]; it is primarily phosphorylated at Ser63 by CK1, which protects it from cleavage by caspase 3 between Asp61 and Asp64 and generates an ~ 28-kDa C-terminal fragment. Thus, upregulation of CK1 may underlie AD pathology by modulating the phosphorylation state of AD-related proteins [115]. In addition, the sAPPβ ectodomain is phosphorylated by CK1 at Ser206 during secretory cleavage [116], while Aβ in turn stimulates the kinase activity of CK1 [117].

NFTs are another characteristic of AD. In the normal state, tau is dephosphorylated and binds microtubules; hyperphosphorylation by CDK5 and GSK-3β inhibits its microtubule-binding capacity, resulting in the release of tau from axonal microtubules into the cytosol, with a consequent reduction in its solubility and microtubule destabilization [109, 118]. Tau oligomerization leads to the formation of NFTs and neuronal apoptosis [119]. CK1 isoforms also contribute to the hyperphosphorylation of tau, leading to its conversion to an abnormal AD-like state [120]. CK1α was found to be closely associated with paired helical filaments (PHFs) purified from the brain tissue of AD patients. Thus, CK1α is one of the major kinases responsible for the pathological hyperphosphorylation of tau protein [121].

Parkinson’s disease (PD) is the second most common late-onset neurodegenerative disease after AD and is characterized by an accumulation of α-synuclein—also known as Parkinson disease protein 1 (PARK1)—and mitochondrial dysfunction [122] as well as bradykinesia, rigidity, and tremor due to the loss of dopaminergic neurons in the substantia nigra [123]. Other pathological hallmarks include progressive neuronal loss in a subset of brainstem and mesencephalic nuclei and aggregation of α-synuclein in the form of Lewy bodies and neurites [124].

α-Synuclein phosphorylated at Ser87 and especially Ser129 is the predominant form of the protein in Lewy bodies [125]. CK1s (mainly CK1α) and CK2 phosphorylate α-synuclein at both residues [126, 127]. CREB, a transcription factor that induces the expression of peroxisome proliferator-activated receptor gamma coactivator-1α (PGC-1α) and confers protection to dopaminergic neurons, is also phosphorylated by CK1α at Ser108/111/114 [128], which may be critical for CRE-mediated gene expression induced by dopamine and calcium [129].

Mutations in PARK proteins (PARK1–PARK8)—especially α-synuclein, Parkin (also known as PARK2), phosphatase and tensin homolog-induced putative kinase 1 (PARK6), DJ-1 (also known as PARK7), and leucine-rich repeat kinase 2 (LRRK2) (also known as PARK8)—have been detected in both familial and sporadic PD [107, 130]. LRRK2 is phosphorylated by CK1α at Ser910/935/955/973 [131], whereas Parkin is phosphorylated by CK1 at Ser101/378 under okadaic acid treatment [132].

**Fig. 9:**
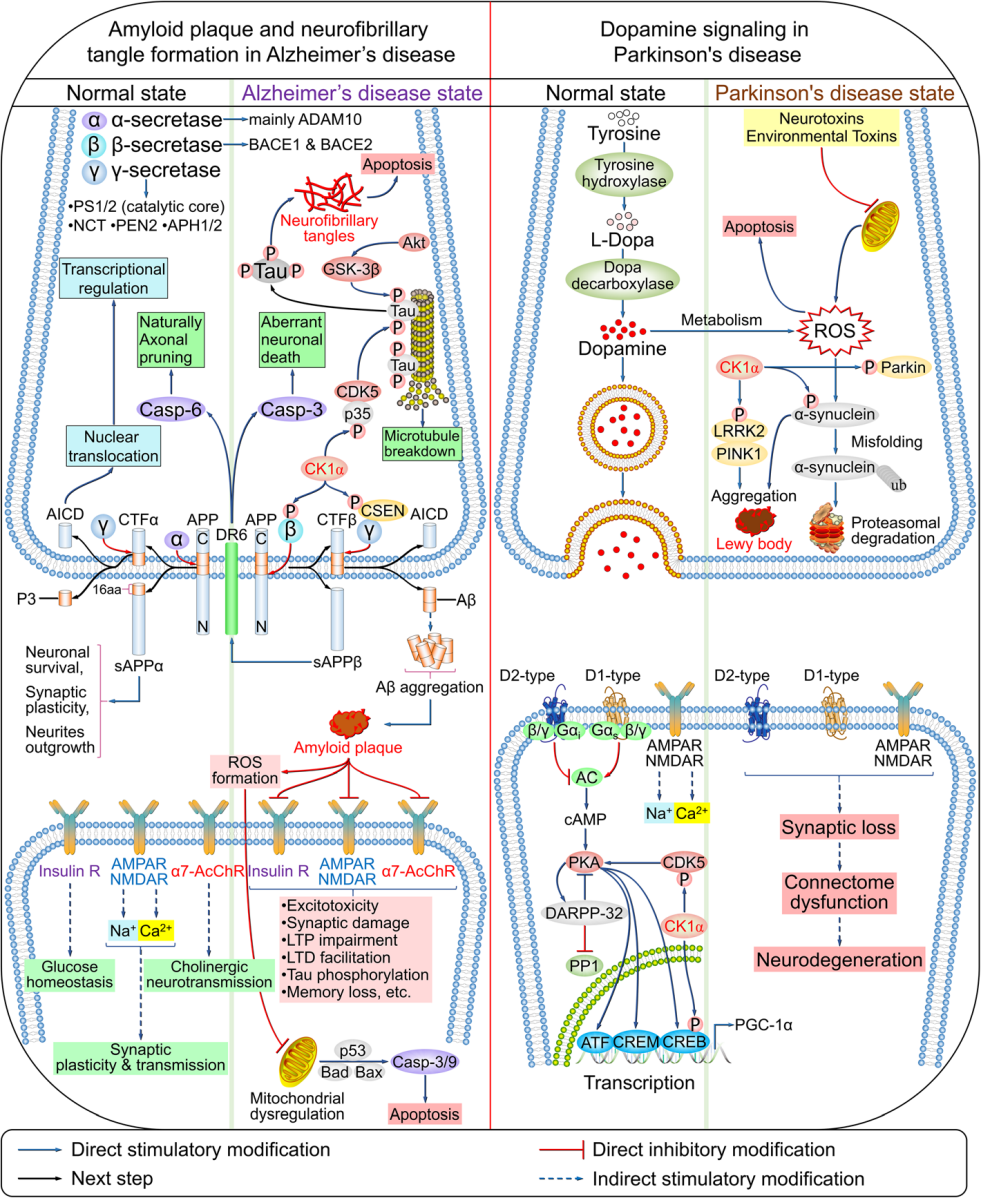
Signaling pathways regulated by CK1α in neurodegenerative diseases. (also reviewed in references [109, 110, 118, 124, 258, 259])

CDK5 is implicated in both AD and PD [133]. CDK5 is phosphorylated by CK1δ at Ser159 [134], whereas p35—the catalytic and regulatory subunit of CDK5—is phosphorylated by CK1α. Additionally, CK1α controls metabotropic glutamate receptor (mGluR)-mediated Ca^2+^ currents in the CK1α/CDK5/dopamine- and cAMP-regulated neuronal phosphoprotein 32 cascade [135]. A recent genome-wide analysis identified *CSNK1A1* as a gene linked to language impairment [136]. Thus, CK1α plays an important role in the pathogenesis of AD and PD (Fig. 9 and Table 1).

### CK1α in the host defense response

In addition to NF-κB signaling, CK1α is also involved in the host defense response against infectious pathogens. CK1α phosphorylates type I interferon receptor 1 (IFNAR1) at Ser535 and thereby induces its ubiquitination and degradation via recruitment of β-TrCP E3 ubiquitin ligase in response to endoplasmic reticulum stress as well as infection [137, 138] by the protozoan *Leishmania major* or vesicular stomatitis virus (VSV) in human cells [137] and by infectious bursal disease virus in chicken [139]. Newly research have demonstrated that CK1α mediates degradation of IFNAR1 and type II IFN (IFN-γ) receptor 1 (IFNGR1) caused by hemagglutinin of influenza A virus (IAV) [140]. CK1α also acts as a specific host factor and is required for the spread of *Listeria monocytogenes* between cells, which occurs via formation of productive membrane protrusions [141]. In *Toxoplasma gondii*, CK1α is essential for replication in host cells; loss of CK1α enhances the virulence of *T. gondii* in mice via upregulation of rhoptry proteins (ROPs), activation of signal transducer and activator of transcription 3, and suppression of IL-12 production [142].

CK1α phosphorylates rotavirus non-structural protein 5 at Ser67 [143]; the hyperphosphorylated form of the protein is required for rotavirus RNA replication [144]. Similarly, CK1α phosphorylates non-structural protein 5A (NS5A) of hepatitis C virus (HCV) at Ser232 and NS5 of yellow fever virus (YFV) at Ser56, leading to hyperphosphorylation of NS5A [145, 146] and NS5 [147] for RNA replication. Thus, CK1α is required for pathogen infection, and specifically for viral RNA replication (Table 2).

**Table 2:** Substrates of human CK1α in various biological events

Gene	Protein	Phosphorylation site	Function	Reference
IFNAR1	IFNAR1	S535	*Leishmania major*/VSV/ IAV	[137, 138, 140]
IFNGR1	IFNGR1	N/A	IAV	[140]
NS5A	NS5A	S232	HCV	[145, 146]
NS5	NS5	S56	YFV	[147]
NSP5	NSP5	S67	Rotavirus	[143, 144]
HNRNPC	hnRNP C1/C2	S240/253, S247/260, S286/S299	mRNA metabolism	[183]
TUT1	Star-PAP, RBM21	S6		[184]
AGO2	AGO2	S824, S828, T830, S831, S834	MiRNA-mediated silencing of target mRNA	[185]
KDM1A	LSD1	S687	Glioblastoma	[173]
PHLPP1	PHLPP1	S1359, T1363, S1379, S1381	Colorectal cancer	[166]
RAPGEF2	RAPGEF2	S1244, S1248	Cancer metastasis	[174]
RXRA	RXRα	N/A	Cancer apoptosis	[175]
Bid	Bid	N/A		[176]

### CK1α in cancer

CK1α is a component of the Wnt/β-catenin signaling pathway that functions as a tumor suppressor [148]. Low levels of *CSNK1A1* may contribute to tumorigenesis and poor prognosis, especially in colorectal cancer according to the data from open-source databases. However, nearest research reported that *CSNK1A1* overexpression correlates with poor survival in colorectal cancer [149]. The opposite conclusions both lack the protein data. Notably, the *P* value of overall survival calculated by Kaplan-Meier method that divided according to relative *CSNK1A1* RNA expression in tumor tissue are both very close to 0.05. Thus, the opposite conclusions need a large sample approach based on protein data for final verdict. CK1α interacts with MDMX to inhibit the DNA-binding and transcriptional activity of p53 [8, 9, 96], resulting in p53 ubiquitination and degradation via interaction with MDM2 [7]. *CSNK1A1* was unrelated to the survival of sporadic colon cancer patients with functional p53, but those with low *CSNK1A1* expression had very poor prognosis compared to patients with high *CSNK1A1* levels and non-functional p53 [150]. Loss of CK1α does not lead to colorectal cancer due to induction of p53, unless both p53 and CK1α genes are deleted [10]. CK1α ablation also leads to activation of the IFN signaling pathway, which prevents unlimited proliferation of intestinal epithelial cells even when β-catenin is constitutively active. Concurrent loss of CK1α and IFNAR1 leads to intestinal hyperplasia, inhibition of apoptosis, and rapid and lethal loss of the intestinal barrier function [151]. Thus, CK1α maintains a balance among Wnt/β-catenin, p53, and IFN signaling. It is also implicated in RAS-driven cancers such as colon cancer—which depends on autophagy [72]—and acts as a negative regulator in prostate cancer [94], liposarcoma [152], and ultraviolet radiation-induced skin tumors [153].

*CSNK1A1* is located on chromosome 5q32 and is downregulated [154] or mutated [155, 156] in patients with in MDS del(5q). *CSNK1A1* mutations have also been detected in adult T cell leukemia/lymphoma (ATL) [157], clear cell renal cell carcinoma [158], colon cancer [159], and esophageal adenocarcinoma [160, 161]. Haploinsufficiency of *CSNK1A1* leads to β-catenin activation and expansion of the HSC pool, whereas homozygous deletion leads to inhibition of HSC proliferation [104]. The observation that over 50% of patients treated with lenalidomide experienced remission [162–164] was attributable to the fact that *CSNK1A1* haploinsufficiency heightens sensitivity to the effects of lenalidomide-induced CK1α degradation [12], which was shown to be mediated by valosin-containing protein (VCP)/p97 [165].

CK1α phosphorylates pleckstrin homology domain leucine-rich repeat protein phosphatase 1 (PHLPP1) at Ser1359, Thr1363, Ser1379, and Ser1381 leading to its ubiquitination and degradation, which may promote colon cancer progression [166]. It also interacts with hematopoietic pre-B cell leukemia transcription factor-interacting protein (HPIP) to stimulate renal cell carcinoma growth and metastasis via activation of mTOR signaling [167]. CK1α is more highly expressed in and can serve as a diagnostic marker for malignant melanoma [168]; however, CK1α suppression in melanoma cells causes a switch in β-catenin signaling to promote metastasis [169, 170]. It is also highly expressed in multiple myeloma and plasma cell leukemia [171], and has an oncogenic role in these malignancies. Likewise, ABC DLBCL requires CK1α for constitutive NF-κB activity and survival; lenalidomide may have therapeutic effects in ABC DLBCL by inducing the degradation of CK1α [11, 12, 172], as well as in pancreatic cancer in which CK1α is upregulated. The current evidence suggests that CK1α dependency resembles non-oncogenic addiction in which the cancer cell phenotype depends on hyperactivation of specific genes including NF-κB [11].

GSK-3β phosphorylates lysine-specific histone demethylase 1A (KDM1A, also known as LSD1) at Ser683 after priming phosphorylation at Ser687 by CK1α. This leads to KDM1A deubiquitination by ubiquitin-specific protease 22 (USP22) and subsequent stabilization, which is essential for glioblastoma development [173]. IKKβ stimulates the CK1α-mediated degradation of Rap guanine exchange factor 2 (RAPGEF2) via phosphorylation at Ser1244 and Ser1248 in response to hepatocyte growth factor (HGF), and may promote the dissemination and metastasis of human breast cancer cells [174].

CK1α interacts with retinoid X receptor α (RXRα) and enhances cell survival by preventing RXR agonist-induced apoptosis in cancer cells [175]. CK1α exerts an anti-apoptotic function by phosphorylating and preventing the caspase-8 dependent cleavage of BH3-interacting domain death agonist (Bid) in HeLa cells [176] (Table 2).

CK1α has also been implicated in lung [80, 148, 177–179], breast [180], esophageal [181], and urothelial [182] cancers. It was found to promote KRASG12D-induced lung cancer through phosphorylation of FADD at Ser194 [80]; CK1α inhibition prevented acquired drug resistance to erlotinib in epidermal growth factor receptor-mutant NSCLC [179]. On the other hand, the Ki-67-interacting protein Nucleolar protein interacting with the FHA domain of pKi-67 (NIFK) enhanced Ki-67-dependent cell migration and invasion in vitro and metastasis in vivo by reducing CK1α level in lung cancer [148]. Thus, CK1α is a potential therapeutic target due to its role as a conditionally essential malignancy protein.

### CK1α in other biological events

The regulation of mRNA metabolism by CK1α is evidenced by its localization at nuclear speckles and roles in the modification of small nuclear ribonucleoprotein particles (snRNPs) [22] and phosphorylation of heterogeneous nuclear ribonucleoprotein C1/C2 (hnRNP C1/C2)—a nuclear-restricted pre-mRNA-binding protein—at Ser240/253, Ser247/260, and Ser-286/S299, which modulates its mRNA-binding capacity [183]. CK1α phosphorylates speckle-targeted phosphatidylinositol-4, 5-biphosphate K1A-regulated poly(A) polymerase at Ser6 and induces the transcription of hemeoxygenase 1 (HO-1) and NAD(P)H quinone dehydrogenase 1 (NQO1) [184]. It was also shown to phosphorylate argonaute 2 (AGO2) at Ser824-Ser834 (mainly at Ser828), thereby preventing AGO2-associated target mRNA binding and attenuating micro (mi)RNA-mediated gene silencing [185]. Systems biology approaches have also identified CK1α as a regulator of the DNA damage response in embryonic stem cells [186].

CK1α-mediated Wnt/β-catenin signaling is essential for ontogenesis and stem cell fate determination [187]; for instance, its ablation causes the naked cuticle phenotype in *Drosophila* [188]. Stromal cell derived factor 1α (SDF1α) inhibits CK1α and attenuates CK1α-mediated phosphorylation, destabilization, and degradation of β-catenin, which is important for c-kit+ cardiac stem/progenitor cell (CSPCs) quiescence under normal conditions and for myocardial regeneration following stress or injury [189]. CK1α suppression leads to Wnt activation and transforming growth factor β/mothers against decapentaplegic homolog 2 inhibition, resulting in the conversion of epiblast stem cells into embryonic stem cells (ESCs) [190] and promoting the establishment and maintenance of the pluripotency network [191]. CK1α directly phosphorylates protein arginine methyltransferase 1 (PRMT1) (mainly at Ser284/Thr285/Ser286/289) to suppress grainyhead-like transcription factor 3 (GRHL3)-mediated terminal differentiation and maintain somatic tissue in a state of self-renewal [192]. Additionally, competitive bone marrow repopulation assays have demonstrated that CK1α is essential for long-term HSCs function [193].

Muscarinic acetylcholine receptors (mAChRs) including M1 [194] and M3 [195, 196] are G protein-coupled receptors (GPCRs) [197] that are phosphorylated by CK1α in an agonist-dependent manner. Phosphorylation of adaptor protein 3 (AP3) by CK1α is required for the efficient formation synaptic vesicles from endosomes [198]. CK1α-mediated phosphorylation stimulates the degradation of the clock protein period circadian regulator 1 (PER1), suggesting a function in circadian rhythm [199]. Mice with heterozygous and homozygous CK1α mutations in the adipose lineage developed diabetes as a result of dysregulated glucose metabolism [200]. CK1α also participates in the regulation of human erythrocyte apoptosis by modulating cytosolic Ca^2+^ activity [201], and promotes homolog pairing and genome organization by inducing the degradation of chromosome-associated protein H2 (Cap-H2) and limiting chromatin-bound Cap-H2 levels in *Drosophila* [202].

### Regulation of CK1α by endogenous factors

CK1α functions as a broad Ser/Thr kinase that regulates multiple biological processes (Tables 1 and 2) and is itself regulated by various factors. For example, the miRNA miR-155 binds to the 3′-untranslated region (3’-UTR) of CK1α mRNA, thereby enhancing Wnt/β-catenin signaling and cyclin D1 expression and promoting liposarcoma cell growth [152]. MiR-155 is also upregulated in systemic and localized scleroderma and may contribute to disease etiology by repressing CK1α and Src homology 2-containing inositol phosphatase 1 (SHIP-1) [203]. Similarly, miR-9-5p binds to the 3’-UTR of both CK1α and GSK-3β, which mediate the migration of mesenchymal stem cells (MSCs) via Wnt/β-catenin signaling [204].

CK1α regulation at the protein level mostly involves transport and subcellular localization, activation/inactivation, and degradation. As stated earlier, CK1α is localized at nuclear speckles and regulates multiple aspects of mRNA metabolism [22, 183]. However, the mechanism underlying CK1α nuclear transport was only recently elucidated: SON DNA-binding protein localizes to nuclear speckles and acts as a scaffold to which CK1α is recruited by family with sequence similarity 83 member H (FAM83H) [205]. Additionally, GLIPR1-mediated redistribution of CK1α from the Golgi apparatus to the cytoplasm as well increased CK1α protein level is essential for β-catenin phosphorylation and destruction [94]. CK1 members were considered as rogue kinases because their enzymatic activity is apparently unregulated. Of note, RNA helicase DDX3 was identified as a binding protein of CK1α which directly stimulates its kinase activity in a Wnt-dependent manner [206]. But no endogenous inhibitor of CK1α has been identified to date, even the degradation of CK1α is mediated by lenalidomide [12, 13, 207].

### Small molecules targeting CK1α

Small molecules are the most useful research tools for investigating protein function, since the clinical application of RNAi and clustered regularly interspaced short palindromic repeats (CRISPR)/CRISPR-associated protein-9 nuclease-mediated gene knockout—while attractive approaches—has numerous challenges or is unfeasible. CKI-7—the first CK1 inhibitor to be developed [208]—is now widely used, with a 50% inhibitory concentration (IC50) of 113–236 μM [80, 209, 210]. IC261 was originally used as a selective inhibitor of CK1ε/δ [211], but has since been shown to block the activity of all CK1 isoforms, with an IC50 of 0.19 μM for CK1α [131, 212]. TG003 was originally identified as a cell division cycle-like kinase inhibitor [213] that suppresses CK1δ/ε activity to a degree equal to or greater than IC261 [214, 215], with an IC50 of 0.33 μM for CK1α [212]. D4476 is the most effective and widely used inhibitor of CK1s, with an IC50 of 200–300 nM [216]. Triamterene—a drug approved by the Food and Drug Administration of the United States (FDA) for the treatment of edematous disorders such as cardiac failure, nephrotic syndrome, and hepatic cirrhosis [217]—was shown to induce epiblast stem cell reprogramming by inhibiting CK1α, with an IC50 of 33.5 μM. However, it also suppressed the kinase activity of CK1δ and CK1ε, with IC50 values of 6.9 and 30.4 μM, respectively [190]. Epiblastin A is a triamterene analog that was developed for more potent inhibition of CK1α; the IC50 values for CK1α, CK1δ, and CK1ε are 3.8, 0.8, and 3.7 μM, respectively [190]. A high-throughput chemical screen identified longdaysin as a small molecule that directly binds CK1α and blocks CK1α-mediated phosphorylation and degradation of PER1, inhibiting CK1α and CK1δ with IC50 values of 5.6 and 8.8 μM, respectively [199].

At present there are no inhibitors that selectively target CK1α or other CK1 isoforms. Nonetheless, the available compounds have been used to study CK1α function. For example, IC261 was used to inhibit CK1α phosphorylation of LRRK2 at Ser935 [131]. In another study, IC261 could not block FADD phosphorylation of FADD at Ser194 by CK1α, although this was achieved by CKI-7 and D4476 [86].

Lenalidomide is a thalidomide analog and FDA approved drug that does not inhibit CK1α but induces CK1α ubiquitination and degradation via CRL4CRBN E3 ubiquitin ligase at concentrations of 0.1–10 μM [12], which has been confirmed by structural analyses [13].

**Fig. 10:**
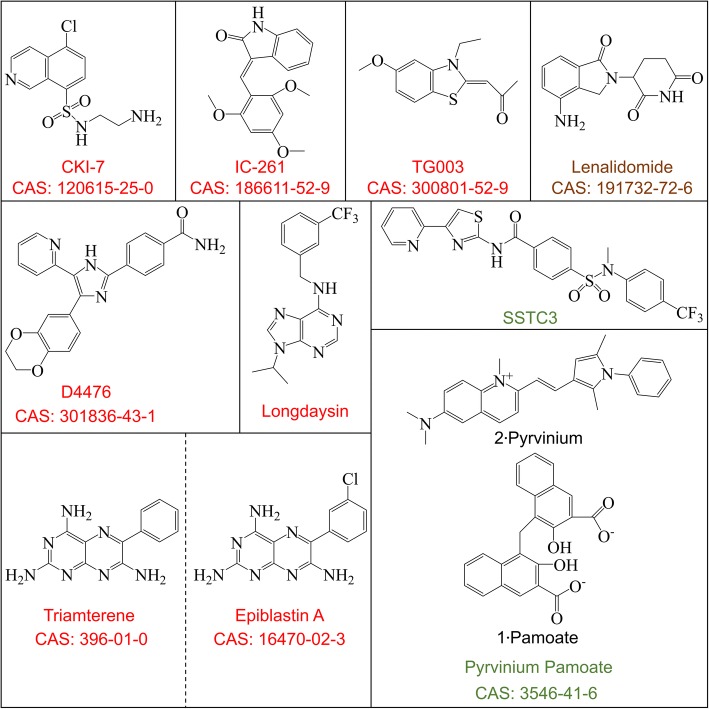
Small molecule inhibitors and agonists of CK1α

Pyrvinium is an FDA-approved antihelminthic drug that has now been replaced by a more effective, broad-spectrum alternative, although it is still available under the Parke-Davis label in Europe and under the name pamoxan (Sato Pharmaceutical, Tokyo) in Japan [218]. Pyrvinium is a potent inhibitor of Wnt signaling that potentiates the kinase activity of CK1α and stabilizes Axin [51]. Oral administration of pyrvinium was shown to attenuate the expression of Wnt signaling targets and prevent adenoma formation in APC^min^ mice [219], in addition to stimulating wound repair and myocardial remodeling [220]. Remarkably, subsequent study indicated that pyrvinium did not activates CK1α, but activated GSK3 and down-regulated Akt signaling pathway. However, the study lacks the evidence such as direct interaction between pyrvinium and GSK3 or Akt [221]. SSTC-104 is a functional analog of pyrvinium that activates CK1α, and may be able to counter aberrant Wnt/β-catenin activation by synovial sarcoma (SS) translocation–SSX (also known as SS18-SSX) fusion protein [222]. Later studies reported that poor bioavailability limited the applicability of pyrvinium, and the new CK1α activator SSTC3—which has better pharmacokinetic properties—was developed [223, 224] (Fig. 10). Interestingly, the histone deacetylase 6 inhibitor ACY-1215 was shown to increase Lys49 acetylation and Ser45 phosphorylation by CK1α without affecting Ser33/37 and Thr-41 phosphorylation by GSK-3β [225].

## Conclusions

Human CK1α is an important protein implicated in colorectal cancer [10], MDS del(5q) [12, 13], ABC DLBCL [11], and neurodegenerative diseases [113, 126, 128, 132]. However, there are many open questions regarding the physiological function of CK1α. Firstly, the mechanism of CK1α regulation remains obscure. At the level of transcription, it is unknown whether the regulatory mechanism involves methylation/demethylation of the *CSNK1A1* gene promoter. At the post-transcriptional level, a few miRNAs such as miR-155 and -9-5p are known to negatively regulate the *CSNK1A1* transcript [152, 203, 204]; however, it is possible that other as-yet unidentified non-coding (nc)RNAs including small nuclear RNAs (snRNAs), small nucleolar RNAs (snoRNAs), long ncRNAs (lncRNAs), and circular RNAs are also involved. CK1α protein expression is controlled at the level of degradation [12, 13] and transport [205]. Although upregulation of PIP_2_ in the plasma membrane was shown to reduce CK1α activity in erythrocytes and neuronal cells [20, 226–228], there is little known about the endogenous mechanisms of CK1α activation/inactivation. As above mentioned, DDX3 directly stimulates the kinase activity of CK1α in a Wnt-dependent manner [206]. A study of CK1α isoforms in zebrafish (*Danio rerio*) suggested that the protein kinase activity of CK1α depends on autophosphorylation of C-terminal residues [229]. Clarifying the mechanisms underlying the activation/inactivation of CK1α in different contexts could provide a basis for designing highly targeted and more effective drugs.

CK1α was recently reported that CK1α participates in p53-dependent paracrine factor secretion in skin hyperpigmentation [230]. Future studies will likely provide additional evidence of a role for CK1α in secretion. In addition, downregulation of CK1α in lung cancer, which induced by NIFK is associated with worse prognosis possibly due to activation of Wnt/β-catenin signaling and stimulation of tumorigenesis [148]. On the other hand, the overexpression of CK1α in other malignancies such as pancreatic cancer has also been linked to poor outcome. Whether CK1α induces constitutive activation of NF-κB in pancreatic cancer as in the case of ABC DLBCL, and how it maintains a balance between Wnt/β-catenin, NF-κB, and other signaling pathways remains to be determined.

Splice variants (isoforms) of CK1α have been identified in cell/animal models such as chicken [231], rat [232] and human [233]. All isoforms of CK1α have CK1 catalytic properties, but exhibit different binding activity toward common CK1 substrates [232]. The different isoforms of human CK1α have variable amino acid sequences and distinct functions. CK1α isoform 1 with an NLS in the 28-amino-acid “L” insert (CK1αLS)—but not isoforms 2–4—regulates nuclear signaling in response to H_2_O_2_ [14]. CK1αLS also promotes vascular cell proliferation and intimal hyperplasia [234], and mediates the effects of NADPH oxidase on vascular activation [235]. The 12-amino-acid “S” insert near the C terminus may function as a kinase domain for CK1α in zebrafish [229]. A phosphoproteome analysis revealed that isoform 2 of CK1α is phosphorylated at Thr321 [236], which may be linked to endogenous activation/ inactivation of CK1α.

Del(5q) can be detected in not only MDS but also acute lymphoblastic leukemia, especially at 5q32 where the *CSNK1A1* gene exists [237]. Thus, CK1α is an attractive molecular target for both diagnosis and monitoring therapy under the treatment of lenalidomide. CK1α is a Ser/Thr kinase with a large number of substrates, some of which have yet to be experimentally verified using approaches such as a pull-down assay, protein interaction domain mapping, and point mutation. A combination of tandem affinity purification and mass spectrometry may facilitate the discovery of new substrates. Additionally, identifying or designing more effective and specific inhibitors, agonists and blocking peptides [95] should enable CK1α targeting in a variety of clinical contexts. Application of small molecule library such as Pfizer compounds and molecular docking algorithm based on the structural information of CK1α may be the most effective approaches so far. Once these inhibitors,agonists and blocking peptides are identified, development of therapy specifically targeting CK1α should open the new avenues for effective management of a broad spectrum of diseases.

## Abbreviations

3’-UTR: 3′-untranslated region
ABC DLBCL: Activated B cell-like subtype of diffuse large B-cell lymphoma
AD: Alzheimer’s disease
ADAM10: A disintegrase and metalloprotease 10
AGO2: Argonaute 2
AICD: APP intracellular domain
AP3: Adaptor protein 3
APC: Adenomatous polyposis coli
APH1/2: Anterior pharynx defective 1/2
APP: Aβ precursor protein
Aβ: Amyloid-β
BCL10: B-cell chronic lymphocytic leukemia/lymphoma 10
BCR: B cell receptor
Bid: BH3-interacting domain death agonist
Brg-1: BRM/SWI2-related gene 1
Cap-H2: Chromosome-associated protein H2
CARD11: Caspase recruitment domain family member 11
CBM: CARD11/BCL10/MALT1
CBP: CREB binding protein
CDC25A: Cell division cycle 25 homolog A
CDK4/6: Cyclin D-cyclin-dependent kinase 4/6
CHR: Cell cycle genes homology region
CK1α: Casein kinase 1α
CKIs: Cyclin-dependent-kinase inhibitors
CRBN: Cullin 4/really interesting new gene-box 1/DNA damage-binding protein 1/cereblon (also known as CRL4^CRBN^)
CREB: Cyclic (c)AMP response element-binding protein
CRISPR: Clustered regularly interspaced short palindromic repeats
CSEN: Calsenilin
CSPCs: Cardiac stem/progenitor cell
CTFα: C-terminal fragment α
DARRP-32: Dopamine- and cAMP-regulated neuronal phosphoprotein 32
DEPTOR: DEP domain-containing mTOR-interacting protein
ESCs: Embryonic stem cells
FADD: Fas-associated death domain
FAM83H: Family with sequence similarity 83 member H
FBXW7: F-box and WD repeat domain-containing 7
FOXM1: Forkhead box M1
FOXO3A: Forkhead box protein O3A
GABARAPs: Gamma-aminobutyric acid type A receptor-associated proteins
GLIPR1: Glioma pathogenesis-related protein 1
GPCRs: G protein-coupled receptors
GRHL3: Grainyhead-like transcription factor 3
GSK-3β: Glycogen synthase kinase 3β
HCV: Hepatitis C virus
HGF: Hepatocyte growth factor
hnRNP C1/C2: Heterogeneous nuclear ribonucleoprotein C1/C2
HO-1: Hemeoxygenase 1
HPA: The human protein atlas
HSCs: Hematopoietic stem cells
IAV: Influenza A virus
IFNAR1: Interferon receptor 1
IFNGR1: IFN-γ receptor 1
IKK: Inhibitor of κB kinase
IκBs: Inhibitor of κBs (including IκBα, β, and ε)
KDM1A: Lysine-specific histone demethylase 1A (also known as LSD1)
LC3-II: Microtubule-associated protein 1A/1B-light chain 3-II
lncRNA: Long non-coding RNA
LRP6: Low-density lipoprotein receptor-related protein 6
LRRK2: Leucine-rich repeat kinase 2
mAChRs: Muscarinic acetylcholine receptors
MALT1: Mucosa-associated lymphoid tissue lymphoma translocation gene 1
MDM2: Murine double minute clone 2
MDM4: Murine double minute clone 4 (also known as MDMX)
MDS del(5q): Myelodysplastic syndrome with deletion of chromosome 5q
mGluR: Metabotropic glutamate receptor
MSCs: Mesenchymal stem cells
mTOR: Mammalian target of rapamycin
MUVB: Multi-vulval class B
MYT1: Membrane-associated tyrosine/threonine 1 (also known as PKMYT1)
NCT: Nicastrin
NEDD4-1: Neural precursor cell expressed: developmentally down-regulated 4-1
NFTs: Neurofibrillary tangles
NF-κB: Nuclear factor κB
NIFK: FHA domain of pKi-67
NLS: Nuclear localization signal
NQO1: NAD(P)H quinone dehydrogenase 1
NS5A: Non-structural protein 5A
NSCLC: Non-small-cell lung cancer
PARK1: Parkinson disease protein 1
PB1: Polar body 1
PD: Parkinson’s disease
PDB: Protein data bank
PEN2: PS enhancer 2
PER1: Period circadian regulator 1
PGC-1α: Proliferator-activated receptor gamma coactivator-1α
PHFs: Paired helical filaments
PHLPP1: Pleckstrin homology domain leucine-rich repeat protein phosphatase 1
PI3K: Class III phosphatidylinositol-3 kinase
PLK1: polo-like kinase 1
PRMT1: Protein arginine methyltransferase 1
PS1/2: Presenilin 1/2
PTEN: Phosphatase and tensin homolog deleted on chromosome ten
RAPGEF2: Rap guanine exchange factor 2
RIP1: Receptor-interacting serine/threonine kinase 1
RNAi: RNA interference
ROPs: Rhoptry proteins
RXRα: Retinoid X receptor α
SDF1α: Stromal cell derived factor 1α
SHIP-1: Src homology 2-containing inositol phosphatase 1
Smo: Smoothened
snoRNA: Small nucleolar RNA
snRNA: Small nuclear RNA
snRNPs: Small nuclear ribonucleoprotein particles
SQSTM1: Sequestosome 1
TCGA: The cancer genome atlas
TCR: T cell receptor
ULK1/2: Unc-51-like autophagy activating kinase 1
USP22: Ubiquitin-specific protease 22
VCP: Valosin-containing protein
Vps15: Vacuolar protein sorting-associated protein 15
VSV: Vesicular stomatitis virus
WTX: Wilms tumor gene on X chromosome (also known as APC membrane recruitment protein 1)
YFV: Yellow fever virus
β-TrCP: β-transducin repeat-containing E3 ubiquitin protein ligase
